# The Use of Murine Models for Studying Mechanistic Insights of Genomic Instability in Multiple Myeloma

**DOI:** 10.3389/fgene.2019.00740

**Published:** 2019-08-15

**Authors:** Philip Vlummens, Kim De Veirman, Eline Menu, Elke De Bruyne, Fritz Offner, Karin Vanderkerken, Ken Maes

**Affiliations:** ^1^Department of Hematology and Immunology-Myeloma Center Brussels, Vrije Universiteit Brussel, Brussels, Belgium; ^2^Department of Clinical Hematology, Ghent University Hospital, Gent, Belgium

**Keywords:** genomic instability, multiple myeloma, mouse models, drug resistance, DNA damage

## Abstract

Multiple myeloma (MM) is a B-cell malignancy characterized by the accumulation of clonal plasma cells in the bone marrow. In normal plasma cell development, cells undergo programmed DNA breaks and translocations, a process necessary for generation of a wide repertoire of antigen-specific antibodies. This process also makes them vulnerable for the acquisition of chromosomal defects. Well-known examples of these aberrations, already seen at time of MM diagnosis, are hyperdiploidy or the translocations involving the immunoglobulin heavy chain. Over the recent years, however, novel aspects concerning genomic instability and its role in tumor development, disease progression and nascence of refractory disease were identified. As such, genomic instability is becoming a very relevant research topic with the potential identification of novel disease pathways. In this review, we aim to describe recent studies involving murine MM models focusing on the deregulation of processes implicated in genomic instability and their clinical impact. More specifically, we will discuss chromosomal instability, DNA damage and repair responses, development of drug resistance, and recent insights into the study of clonal hierarchy using different murine MM models. Lastly, we will discuss the importance and the use of murine MM models in the pre-clinical evaluation of promising novel therapeutic agents.

## Introduction

Multiple myeloma (MM) is a bone marrow malignancy defined by the presence of atypical clonal plasma cells in the bone marrow. These cells produce a monoclonal protein, also called M-component, which is a derivative of a normal immunoglobulin protein and can be detected using protein electrophoresis on serum and/or urine of the patient. Symptomatic MM disease is typically preceded by a precursor state termed monoclonal gammopathy of undetermined significance (MGUS) (Kyle et al., 2002). Here, an M-component can be detected but in absence of any end-organ damage. Smouldering MM (SMM) is the intermediate disease stage, positioned between MGUS and MM in the disease evolution. In SMM, end-organ damage is also absent but higher levels of tumor burden are seen alongside a higher risk of progression toward MM (Kyle et al., 2002; Rajkumar et al., 2014). MM accounts for around 1% of all cancers and 10% of all hematological malignancies in Western populations (Moreau et al., 2013; Rajkumar et al., 2014). The treatment of MM has made an enormous evolution the last few years with the development of the so-called novel agents. Introduction of these agents, including proteasome inhibitors (bortezomib, ixazomib, carfilzomib), immunomodulatory agents (thalidomide, lenalidomide, and pomalidomide) together with consolidation using high-dose melphalan followed by autologous stem cell salvage in fit patients, has already led to an immense improvement of overall survival in MM patients. Additionally, introduction of monoclonal antibodies targeting CD38 or SLAMF7 (daratumumab and elotuzumab) or cellular therapy (CAR-T) will most likely further improve treatment-free remission and OS in the MM patient population (Rajkumar et al., 2014; D’Agostino et al., 2017; Zhao et al., 2018). Unfortunately, nearly all patients will eventually relapse and develop refractory disease, wherefore MM is still seen as an incurable disease.

One aspect of the development of relapsed and refractory MM is genomic instability, which is already present at the time of diagnosis and can ultimately lead to clonal evolution and drug resistance (Bolli et al., 2014; Lohr et al., 2014; Walker et al., 2015). This aspect of the disease implicates the fact that the medical need for a more thorough understanding of genomic instability in MM disease alongside novel therapeutic options, countering these processes, remains high. Important tools to study these topics in MM are the different MM murine models, which enable researchers to mimic human MM disease as close as possible in a controlled manner. In this review, we aim to focus on the different types of murine MM models available, their genetic aspects within the scope of genomic instability and the different research fields where these mouse models have been implicated to further our understanding of genomic instability in MM, and in the end, improve the outcome of our patients.

## Description of Mechanistic Insights of Different Murine MM Models

Up till now, different murine MM models have been reported and used in biomedical research, aiming to study the pathological basis of MM disease on the one hand and identification of possible novel agents on the other (**Table 1**). Up to date, four different approaches to establish murine MM models have been described, being models with spontaneous development of MM disease versus models using an induction, transgenic or xenograft approach (Radl et al., 1988; Carrasco et al., 2007; Chesi et al., 2008; Morito et al., 2011). As the mechanism of generation is inherently distinct between the different types of murine models, differences in genomic build and possible correlation with human MM disease are present. As such, the ideal murine MM model, spanning all aspects of disease biology and genetic build, unfortunately does not exist. Each system has specific strengths and weaknesses, which need to be evaluated in view of the scientific question in need of answering.

**Table 1:** Schematic overview of murine models available for studying MM disease biology. Models are categorized according to genetic build.

Model category	Murine MM model	Primary features
Xenograft models	NOD/SCID NOD/SCID/IL2Rgamma	Injection of MM cells (primary patient samples of cell lines) IV or SC. MM disease has pathogenic features of injected material. Requires transplant.
	MIS^(TK)^TRG6	Humanized model based on knock in of human growth factor and cytokine encoding genes. Facilitates multi-lineage engraftment of human immune and hematopoietic stem cells
	SCID-hu	Implantation of human embryonic bone fragments that can be inoculated with human MM cells, lesser used due to ethical issues.
	SCID-rab	Model derived from SCID-hu model, implementing rabbit bone fragments.
	SCID-synth-hu	Model derived from SCID-hu model, implementing polymeric scaffold coated with human bone marrow stromal cells.
Immunocompetent spontaneous models	5TMM models	Syngeneic model from different 5TMM strains which developed spontaneously in aged C57BL/KaLwRij mice. Requires transplantation of model for propagation. Extensively characterised genetically.
Immunocompetent transgenic models	Eµ-XBP1	Overexpression of XBP1 isoform involved in MM pathogenesis and government of unfolded/ER stress response.
	Eµ-MAF	Regulation of MAF oncogene by murine immunoglobulin enhancer. Model for human t(14,16) MM disease.
	VK*Myc	Overexpression of Myc in the post-germinal B-cell compartment through AID hypermutation. Indolent disease phenotype. Aggressive phenotype can be generated *via* serial transplantation.
	Myc/Bcl-Xl	Double-transgenic mouse strain overexpressing both Myc and Bcl-Xl.
Induced immunocompetent models	Mineral oil-induced plasmocytoma	Local injection of mineral oil derivatives results in local induction of plasma cell tumor. More representative of plasmocytoma than MM disease. MOPC315.BM model can be transplanted to resemble systemic disease
Single gene KO models	i.e., rrm2 -/-	Mostly generated to study single gene function.

### 5TMM Murine Myeloma Models

The 5T murine MM models are well characterized spontaneous syngeneic immunocompetent MM models. The model is based on the observation by Radl et al. that a small proportion of older animals from the C57BL/KaLwRij inbred strain develop a benign monoclonal gammopathy comparable to the MGUS state in human patients (Radl et al., 1979; Radl et al., 1988; Garrett et al., 1997). As such, these animals have the potential to develop MM or to a lesser degree other B-cell malignancies, such as B-cell non-Hodgkin lymphoma. The 5TMM models were established using serial transplantation of bone marrow and as such closely resemble human disease as it has been shown that interaction with the bone marrow microenvironment is important in disease development, clonal selection, and genomic instability. When transplanted into syngeneic mice, recipients develop the presence of a monoclonal protein and osteolytic bone disease, inevitably leading to hind limb paralysis (Manning et al., 1992; Vanderkerken et al., 2000; Vanderkerken et al., 2003). The 5T33MM and 5T2MM models are the most characterized and widely used models of the 5TMM series. From the 5T33MM model, two clonally related *in vitro* growing cell lines were derived which are stroma independent, being the 5T33vt and 5TGM1 cells. These models represent a single MM clone. It is important that these models have an intact immune system, a feature that is an important advantage when compared with immunocompromised MM animal models. Over the years, these models have been used extensively to study the different aspects of MM pathobiology.

### Transgenic Murine MM Models

Other widely used murine MM models are the transgenic models, which involve overexpression of known MM oncogenes under control of an immunoglobulin enhancer. Three different models have been reported up to date. The Eµ-XBP1s murine transgenic model involves overexpression of the XBP-1 spliced isoform, necessary for the government of the unfolded protein/ER stress response and plasma cell development (Carrasco et al., 2007). These mice have been shown to develop an MGUS/MM phenotype, and gene expression profiling revealed enriched activity of pathways involved in hyperproliferation, IL-6 activation alongside dysregulation of genes known to be driven by recurrent chromosomal translocations in human MM disease. The Eµ-MAF model adopts a similar approach and involves regulation of c-MAF by the immunoglobulin enhancer in the murine B-cell compartment (Morito et al., 2011). As such, transgenic Eµ-MAF mice serve as a model for human MM disease harboring t(14;16), which is known to lead to an aggressive MM phenotype and shorter survival. Thorough insights in the genetic instability in this model are, however, absent. The Vk*MYC model is a third transgenic model, in which the MYC oncogene becomes overexpressed in the post-germinal B-cell compartment *via* AID-dependent somatic hypermutation (Chesi et al., 2008). This model has been shown to give rise to a murine MM phenotype similar to human MM disease with an indolent phenotype at onset of disease, as such following the natural history of MM disease in humans to a great degree (Chesi et al., 2008; Chesi et al., 2012). An aggressive phenotype can, however, be generated using serial transplantation and can be a model for studying relapsed MM (Chesi et al., 2012). This model has already been extensively used for research into the pathogenesis and pre-clinical drug development (Chesi et al., 2012; Matthews et al., 2013). A final transgenic murine MM model, which also focuses on overexpression of MYC oncogene is the Myc/Bcl-X_L_ model (Cheung et al., 2004). This model was generated by creating a double-transgenic Myc/Bcl-X_L_ mouse strain shown to develop plasma cell tumors with an average onset of 135 days at 100% tumor penetrance. The mice developed a monoclonal protein and displayed infiltration of the bone marrow by plasma cells.

### Xenograft Murine MM Models

Xenograft models are immune incompetent murine MM models and are based on the engraftment of primary human MM cells or MM cell lines in immune compromised mice, such as severe combined immunodeficient (SCID), nude or non-obese diabetic (NOD)/SCID or NOD/SCID/IL2Rgamma mice. This model can be used to generate systemic or local disease through intravenous (IV) or local injection of MM cells, respectively. This approach has the advantage that it enables the study of homing of MM cells and to test novel therapeutic compounds in human MM cells *in vivo* (Mitsiades et al., 2003). Currently, several xenograft murine MM models exist, generated using the IV injection of primary patient samples or human MM cell lines, such as OPM2, JJN3, MM.1S, XG1, KMM-1, and RPMI-8266 (Paton‐Hough et al., 2015). A relative drawback of these models is, however, the fact that the majority of human MM cell lines are primarily derived from primary human plasma cell leukemia samples which lack dependency on the bone marrow microenvironment. As such, human MM cell lines could be less representative of the more indolent course of a significant proportion of MM disease. Nevertheless, thorough genetic characterization of human MM cell lines has shown that MM cell lines cover the spectrum of molecular heterogeneity seen in patients, validating them as important tools for studying MM (Moreaux et al., 2011). Using primary patient samples alleviates this aspect but has implications on experimental setup due to patient variability. Recently, the MIS^(KI)^TRG6 murine MM model has been reported as a possible mouse model to study human cells in mice more easily. The model was generated by human IL-6 knock-in modification of MIS^(KI)^TRG mice, which were established by knocking in genes encoding human M-CSF, IL-3, GM-CSF, TPO, and SIRPα into the respective murine loci in immunodeficient Rag2^-/-^ IL2Rgamma ^-/-^ mice. MIS^(KI)^TRG mice were shown to overcome the lack of inter-species cross-reactivity of these cytokines and growth factors to facilitate multi-lineage engraftment of human immune and hematopoietic stem cells (Rongvaux et al., 2014; Das et al., 2017). Subsequent knock-in of human IL-6, an important growth factor for human MM cells, generated an immunodeficient mouse strain in which cells derived from patients with MM or precursor lesions could be xenografted successfully and subsequently used for biomedical research (Das et al., 2017). SCID-based xenograft models that are less widely used today include the SCID-hu model. This model involves the implantation of human fetal bone subcutaneously. Afterward, human MM cells, both cell lines or primary patient material, can be injected directly into the engrafted bone. These mice will develop a spectrum of human MM characteristics, including bone disease and the presence of a paraprotein in the serum (Urashima et al., 1997; Yaccoby et al., 1998; Tassone et al., 2005). Although this model was characterized by a high level of tumor engraftment, important ethical issues concerning the use of human embryonic material led to the abandonment of this murine model. Derivatives of this model include the SCID-rab model, in which rabbit bones are implanted in replacement of human embryonic bone chips, and the SCID-synth-hu model, implementing a 3D polymeric scaffold coated with human bone marrow stromal cells (Yata and Yaccoby, 2004; Calimeri et al., 2011). Both models have the benefit that they overcome the ethical issues raised with the original SCID-hu model and have been implemented to study local MM disease (Yaccoby et al., 2007; Pennisi et al., 2009; Calimeri et al., 2011). Due to the nature of the bone material used, results cannot be readily extrapolated to human disease. Nowadays, the previously discussed MIS^(KI)^TRG6 model can, however, act as a full-fledged replacement of the SCID-hu model, due to the specific genetic build (Das et al., 2017).

### Induced and Single Gene Knockout MM Models

Induced syngeneic MM models are immunocompetent and have been developed by using injection of mineral oil derivatives, such as pristane (Potter and Boyce, 1962; Anderson and Potter, 1969). An important remark concerning these models is the fact that they resemble more a solitary plasmacytoma phenotype and not so much MM disease. One such an induced model, the MOPC315.BM model, has been described to be transplanted successfully in syngeneic animals, leading to successful homing of cells to the bone marrow and the axial skeleton (Hofgaard et al., 2012). More rarely, single gene knock-out mouse models are reported to develop plasma cell malignancies (Chang et al., 2013).

## Molecular and Genetic Characterization of MM Murine Models, Focusing on Genomic Instability

Characterization of the different murine MM models must be approached in view of the mode of construction. As such, transgenic murine models are generally driven by the involved transgene, whereas xenograft models are dependent on the genetic build of the injected MM cells. Extensive characterization into the epigenetic deregulation and/or the presence of chromosomal instability in the different models is as such present in variable degree.

In the 5TMM models, our group recently performed an extensive genetic analysis using next-generation sequencing to evaluate copy number alterations (CNA) and the mutational spectrum (Maes et al., 2018). Analysis revealed that the 5T2MM cells contained focal amplifications involving oncogenes, such as Kras, Nras, Map2k2, and Pik3ca. The 5TGM1 and 5T33vv models also had focal Kras and Akt1 amplifications, respectively. These genes belong to the Ras/MapK and PI3K/Akt pathways, known to be involved in genomic instability. Additionally, several of the identified CNAs encompass genes involved in the Fanconi Anemia DNA-repair pathway. Strikingly, a deletion of the tumor suppressor, Brca2, involved in homologous recombination, was seen in all the 5TMM models. We also described an overlap of 66.4% of genes affected by CNAs in gain of 1q in patients and genes affected by CNA in the 5T2 models. A more detailed analysis revealed an overlap of genes (Cks1b, ILf2, Adar, Arnt) described to be drivers of the poor prognostic features of gain(1q), which could be helpful to further explore why gain(1q), a known marker of high-risk disease, leads to a decrease in overall survival in myeloma patients (Shah et al., 2016). Similarly, a remarkable overlap of 69.9% of genes involved in del(13q) in patients and genes with CNAs in the 5T33 and 5TGM1 models was observed. A more detailed analysis confirmed the loss of important tumor suppressor genes including Rb1, Ebpl, and Rnaseh2. Interestingly, regions on chromosome 5 were commonly deleted in all 5TMM models and encompass Brca2, Flt3, Fgfr2, and Whsc1. Recently, a similar observation was reported in the Vk*MYC model using single-cell chromosomal copy number analysis. Copy number analysis showed that a loss of chromosome 5 was observed in the majority of MM cells from tumor bearing mice, where this was not observed in the normal plasma cell compartment. These observations, present both in the 5TMM and the Vk*Myc MM models, could suggest that CNAs of chromosome 5 are an early and highly prevalent event in murine MM pathobiology (Croucher et al., 2018) and show that genomic instability is an inherent trait of these murine MM models. These observations validate their potential use for exploring involved pathways in MM, especially since many of the observed genetic aberrations are known to be linked with poor prognosis in human MM disease (Manier et al., 2017). With respect to the mutational spectrum observed in the 5TMM models, the amount of non-synonymous mutations exceeds the amounts found in MM patients, indicating that the 5TMM models are more advanced stages of MM disease. Nevertheless, it is clear that the mutated genes are implicated in several key pathways known to be deregulated in MM. These include PI3K/Akt, growth factor-related signaling, cell cycle and DNA repair, and extracellular matrix organization. Genes with mutations that overlap with genes mutated in MM patients include homozygous deletion in Rb1 (5T2) and homozygous loss-of-function mutation in Trp53 (5T33, 5TGM1).

## Specific Topics Stressing Importance of Murine Models in MM

Murine MM models are, due to the significant genetic overlap with human disease, ideal models for studying MM pathogenesis and potential novel therapies. Moreover, they prove to be of use to study the concepts of epigenetic deregulation, DNA damage, chromosomal instability, and the subsequent processes of clonal selection and the development of drug resistance.

### Use of Murine MM Models in the Study of Chromosomal Instability

Chromosomal instability (CIN) is a characteristic of cancer cells, not solely limited to B-cell malignancies. It can lead to numerical imbalances and has been described as an early event in MM disease with gain 1q and del 17p being the most well-known examples. Moreover, CIN has been shown to influence survival in MM patients (Carter et al., 2006; Zhou et al., 2013).

Recently, the role of miR-137 in MM, located on the frequently deleted chromosomal locus 1p22 in MM disease, was studied using a murine xenograft model (Yang et al., 2015; Qin et al., 2016). They showed the presence of a significant inhibition of tumor formation without overt toxicity, alongside a significant and positive effect on overall survival. Subsequent analysis of tumor tissues revealed a significant effect on apoptosis with a decrease in MCL-1 and increase in BAD and PARP upon miR-137 overexpression. In a follow-up study, they were subsequently also able to, using a similar xenograft approach, investigate the epigenetic regulation or miR-137 and report effects of miR-137 therapy on CIN in MM. More specifically, the use of a murine MM model enabled them to evaluate the *in vivo* effects of miR-137 overexpression upon bortezomib treatment, showing smaller tumor volumes and an increased sensitivity to treatment due to downregulation of the AURKA/p53/ATM/Chk2 pathway involved in apoptosis and CIN. These observations were corroborated by Shuaishuai et al. (2017) who used a similar xenograft approach to show that AURKA is involved in chromosomal instability and drug resistance through the DNA damage response Atr-Chk1-gammaH2AX pathway. Downregulation of AURKA promoted apoptosis and alleviated drug resistance. As mounting evidence shows that AURKA plays a role in (early) relapse of MM and drug resistance, inhibition of the AURKA pathway, using miRNA-137 modulation or direct inhibition, could prove an interesting therapeutic strategy targeting genomic instability.

Another implementation of murine models for expanding our insights into CIN was provided by Chang et al. (2013). They created a Rrm2b^+/−^ knockout model (C57BL/6J background) and observed that these mice displayed higher levels of IL-6 when compared to wild-type littermates. Whereas Rrm2b^-/-^ mice displayed a lethal phenotype by 7 weeks of age, heterozygous knockout mice showed persistent high levels of IL-6 and began developing infiltration of a CD138+/kappa+ plasma cell population in the bone marrow and splenic compartments, suggestive for the development of a plasma cell dyscrasia. Subsequent molecular analysis revealed that loss of Rrm2b function led to DNA damage through depletion of dNTP pools and activation of NF-KB and STAT3 signaling with persistent IL-6 secretion. Chromosomal analysis revealed the presence of marked chromosomal aberrations in Rrm2b^+/−^ mice.

Other CIN genes that have been implicated in MM and are good examples of showing the contribution of murine MM models to the field are NEK2 and TRIP13. NEK2 was primarily identified as a CIN gene of interest by performing an intersection analysis of differentially expressed genes between paired MM patient samples from diagnosis and after induction chemotherapy on the one hand and paired MM patient samples from time of diagnosis and at early relapse (Zhou et al., 2013). A total of 56 common genes were identified of which 10 genes (TOP2A, CDC20, TRIP13, NEK2, AURKA, RRM2, CCNB1, KIF4A, CEP55, and PBK) had a significant impact on survival and belonged to the CIN signature as reported in the literature (Carter et al., 2006; Zhou et al., 2013). NEK2 is a cell cycle-regulated protein kinase important for regulating correct centrosome separation during the G2 phase, and it also plays a role in activation of the G2 checkpoint (Fletcher et al., 2004). *In vivo* evaluation of NEK2 inhibition was evaluated by using a NOD-rag/null gamma xenograft model. Mice were injected with ARP1 cells transfected with a shRNA targeting NEK2 or an empty vector. Mice bearing NEK2 downregulated-MM tumors displayed a marked inhibition of tumor growth when compared with control mice. Also, NEK2 downregulation in the bortezomib-resistant APR1-DR MM cell line resulted in a re-sensitization of these cells to bortezomib *in vivo*. A second CIN gene that has been validated in MM using murine models is the AAA-ATPase TRIP13, a key regulator of the spindle assembly checkpoint and chromosome structure (Tipton et al., 2012; Ye et al., 2015). Its importance in MM is also further stressed by the fact that it has been identified as part of the 70-gene high risk model of MM, which is linked with a dismal prognosis (Shaughnessy et al., 2007; Heuck et al., 2014). Tao et al. showed the functional importance of TRIP13 in MM pathogenesis using a NOD-RAG/null xenograft model. Mice were injected with OCI-MY5 cells transduced with TRIP13-shRNA or scrambled vectors and TRIP13 knockdown mice clearly showed less tumor burden compared to controls. As TRIP13 is associated with high-risk disease, chromosomal instability, and drug resistance, its use as a biomarker of high-risk disease on the one hand and its use as a potential therapeutic target need to be explored in the future (Tao et al., 2017).

### Drug Resistance, Risk Stratification, and Drug Development

Despite the availability of many novel agents, MM still remains an incurable disease due to the development of drug resistance and, ultimately, the development of refractory disease. It has been recently shown that the development of MM is not solely due to abnormalities of the plasma cells but also due to changes in the bone marrow microenvironment (Bianchi and Munshi, 2015). The MM cells interact with cells of the bone marrow microenvironment through cell surface molecules and as such initiate signaling pathways influencing drug-induced apoptosis and MM cell survival. Also, the relative hypoxic state of the bone marrow microenvironment has been shown to influence MM cell survival and resistance to therapies. Identification of pathways involved in modulating drug resistance is therefore important, because this could lead to identification of possible novel treatment strategies. As there is an interplay between the MM cells and the bone marrow stroma, evaluation of potential new drugs in murine models adds an extra layer of evidence that the observed effects of the compound studied are not abrogated by the presence of MM–BM interactions and that the compounds under study have an acceptable toxicity profile *in vivo*, a prerequisite for further development. Many targets involved in MM drug resistance and the role of the bone marrow environment have already been identified. Here, selected certain targets can lead to novel therapeutic insights or the development of novel compounds and for which murine models have been extensively used.

A gene of interest concerning this topic is FOXM1, coding for the forkhead box M1 transcription factor. It has been shown that overexpression of FOXM1 is upregulated in high-risk MM and is correlated with shorter overall survival (Gu et al., 2015; Gu et al., 2018a). Gu et al. evaluated the role of FOXM1 *in vivo* data using a NOD/SCID/IL2Rgamma CAG xenograft model. Mice were inoculated with CAG cells with either normal or overexpressed FOXM1 and followed for 7 days after which mice were treated with either bortezomib or drug vehicle. Tumor diameters were found to be larger in the control mice injected with cells overexpressing FOXM1 as was comparable with earlier results. In mice treated with bortezomib however, FOXM1 overexpression resulted in a slight decrease in drug sensitivity (Gu et al., 2018b). This finding was compatible with earlier observations showing that downregulation of FOXM1 can enhance bortezomib-induced cell death in solid tumor cell lines (Pandit and Gartel, 2014).

Studying drug resistance using murine models is, however, also extremely interesting in the field of novel drug development. Recently, RRx-001 has been identified as a potential novel therapeutic agent in many malignancies. It is a dinitroazetidine derivative that induces immunomodulatory effects either through polarization of tumor-associated macrophages or *via* increased T-lymphocyte infiltration (Zhao et al., 2017). *In vivo* treatment with this compound using the human MM.1S xenograft mouse model decreased tumor burden and prolonged survival in treated animals, thus generating a proof of concept for clinical studies with RRx-001 (Das et al., 2016).

Mice models also lead to the introduction of the melphalan-flufenamide dipeptide as a potential novel treatment in MM patients (Chauhan et al., 2013). Melphalan is an alkylating agent that is actively used in MM therapy. Unfortunately, treatment is associated with dose-limiting toxicity, as well as the development of resistance (Kumar et al., 2018). Melphalan-flufenamide, also called melflufen, has been shown to enhance the therapeutic potential of melphalan as its prodrug form allows the compound to accumulate intracellularly more rapidly and with higher concentrations. As such, Chauhan et al. evaluated the *in vivo* efficacy of melflufen using the human MM.1S xenograft mouse model. These experiments showed that melflufen was able to inhibit MM cell growth and prolong mice survival. Melflufen-treated mice survived for a statistically significant longer time, identifying melflufen as a promising compound (Chauhan et al., 2013). As such, a current phase 3 trial is currently actively recruiting patients to evaluate the potential use of this drug as a treatment option.

As stated earlier, acquired drug resistance is a critical problem that every patient and healthcare provider has to face during MM treatment. Many second-generation novel agents have become available the last couple of years, but the need for other therapeutic approaches remains high. One of these approaches focuses on tackling drug resistance and spins off from the observation that many types of cancer cells use the transport of tumor-suppressive proteins from the nucleus to the cytoplasm. As such, tumor cells can render them functionally incapacitated and evade anti-cancer mechanisms *via* shuttling of key proteins, such as p53, Rb, or other cell cycle regulators (Turner et al., 2009; Turner et al., 2013). The implication of this mechanism in MM drug resistance was first described by Turner et al. in 2004, showing that MM cells actively export endogenous topoisomerase II alpha (TOP2A) toward the cytoplasm during MM cell growth, due to enhanced activity of the Exportin 1 protein (XPO1 or CRM1) (Turner et al., 2004). Subsequent inhibition of XPO1 function was shown to prevent export of TOP2A and sensitize drug-resistant MM cells to doxorubicin (Turner et al., 2016a). A similar effect was seen when treating primary samples from proteasome inhibitor-refractory MM patients with a combination of selinexor, a clinical XPO1 inhibitor, and bortezomib or carfilzomib (Turner et al., 2016b). XPO1 inhibition was shown to be able to potentially overcome drug resistance in MM, thus warranting further exploration of XPO1 inhibitors both pre-clinical as clinical. *In vivo* use of XPO1 inhibitors showed marked activity in a NOD/SCID mouse model in which both drug-resistant U266PSR and parental U266 cells were injected (Turner et al., 2016b). Mice were subsequently treated with doxorubicin and selinexor both in monotherapy and combination therapy. When compared with doxorubicin monotherapy, combination of doxorubicin with selinexor significantly reduced tumor burden and increased survival in mice injected with both parental and drug-resistant U266 cells, showing that XPO-1 inhibition can overcome previously acquired drug resistance to a certain degree. Of interest, 60% and 40% of combination-treated mice, challenged with parental or drug-resistant U266 cells, respectively, survived at termination of the study (timepoint of 4 months after injection), whereas all untreated control, doxorubicin, or selinexor monotherapy mice had to be euthanatized before the end of the experiment due to development of bulky disease. Toxicity, assessed by weight loss > 10%, was acceptable and seen in 2% of treated mice. Due to these results both *in vitro* and *in vivo*, subsequent trials with selinexor were initiated in human patients both in monotherapy and in combination with standard of care agents with promising phase I/II results. As such, phase III results are eagerly awaited (Bahlis et al., 2018; Vogl et al., 2018). Of note, however, the toxicity profile in these trials was more elaborate when compared with murine studies.

### DNA Damage and Repair Mechanisms and Their Involvement in Drug Development

Part of the mechanism contributing to overall genomic instability in MM can be explained through the deregulation of DNA damage and repair mechanisms. Recent studies state that the ongoing generation of DNA damage, coupled with deregulation of DNA damage repair, induces further generation of genomic changes (Shammas et al., 2009; Walters et al., 2011; Cottini et al., 2015; Herrero et al., 2015). DNA damage repair mechanisms are necessary to limit the potential impact of insults to DNA integrity, such as double-stranded breaks (DSB), and maintain genomic stability and cellular viability (Ciccia and Elledge, 2010). DSBs can be caused by exogenous events, i.e., genotoxic stimuli, or endogenous events, such as replication stress. DSBs are mainly repaired *via* two distinct pathways, being non-homologous end joining repair (NHEJ) and homologous recombination (HR). NHEJ is based on direct ligation of the damaged DNA and is involved in non–replication-associated DSBs. HR involves, on the other hand, processes that tackle DSBs generated during DNA replication (Brandsma and Gent, 2012). As replication stress and DNA damage have been described in subsets of MM patients and infer high-risk disease, further mechanistic insights are needed and could lead to development of novel compounds, treatment schemes, or drug repurposing (Cottini et al., 2015).

A remark that can be made concerning this topic is the fact that there are genes or pathways that are involved both in drug resistance and DNA repair mechanisms (Gourzones-Dmitriev et al., 2013). One of these, High Mobility Group 1 (HMGB1), is of potential interest as it is implicated in MM but also in many other malignant diseases. HMGB1 is a highly conserved gene. The protein acts as a nucleosome stabilizer and plays an important role in gene transcription, spatial arrangement, and DNA repair (Guo et al., 2018; Hong et al., 2019; Zhang et al., 2019). To confirm HMGB1 as a potential target *in vivo*, Guo et al. inoculated NOD-SCID mice with RPMI8226 cells that were transfected with either control or HMGB1 shRNA. One week after injection, mice were randomized to receive dexamethasone treatment or placebo. These experiments showed that HGMB1 knockdown mice had lower tumor burden after dexamethasone treatment. Although no reports concerning OS or toxicity were available, targeting HMGB1 from a therapeutic point of view certainly remains interesting (Guo et al., 2018).

Another possible example of exploiting the DNA damage repair in MM is the use of DNA methyltransferase inhibitors (DNMTi) and histone deacetylase inhibitors (HDACi). DNMTi, such as azacytidine and decitabine, have already been used in different hematological malignancies and one HDACi, panobinostat, has been investigated in clinical trials and is currently reimbursed in patients with RRMM when used in combination with bortezomib and dexamethasone (Jabbour et al., 2017; San-Miguel et al., 2017). We investigated the effect of decitabine treatment both *in vitro* and *in vivo* (Maes et al., 2014). We were able to show that exposure of MM cells to decitabine-induced cytotoxicity *via* DNA damage as higher levels of gamma-H2AX foci were seen alongside cell cycle arrest and caspase induction. Interestingly, combination of DNMTi with the HDACi quisinostat resulted in an enhanced cytotoxic effect. These *in vitro* findings were able to be reproduced in the 5T33MM immunocompetent model. In a decitabine monotherapy experiment, mice were inoculated with 5T33MM cells and were treated with decitabine or placebo from day 1 of injection. Treated mice showed a significant, dose-dependent improvement of overall survival when compared with untreated mice. Proof-of-concept of combining DNMTi with HDACi was also reproducible *in vivo*, as mice treated with a combination of decitabine and quisinostat had a significant longer survival when compared to placebo or mice treated with these compounds in monotherapy.

The increased insights into the DNA damage and repair response lead to the development of EDO-S101. EDO-S101 is a first-in-class alkylating HDACi fusion molecule that combines the DNA damaging effect of bendamustine, a nitrogen mustard derivative known to induce inter- and intra-strand DNA crosslinks, with the pan-HDACi vorinostat. The rationale behind the pre-clinical development of this fusion compound arises from the hypothesis that histone acetylation would induce a more opened chromatin structure that would be more susceptible to the alkylating moiety. *In vitro* treatment of different MM cell lines and primary patients’ samples indeed showed that EDO-S101 has potent anti-myeloma effects, independent of p53 status or melphalan resistance (López-Iglesias et al., 2017). *In vivo* activity and toxicity were subsequently evaluated in two independent murine MM models. CB17-SCID mice were SC injected with MM1.S cells to create an immune-deficient xenograft model and were subsequently treated with EDO-S101 using two strategies. In a first approach, mice were treated from first day onward. Treated mice showed a slower tumor growth alongside a significant improvement in overall survival. Although a 10% to 20% loss of body weight was seen in treated mice, a spontaneous recovery was seen after the treatment. In a second study, CB17-SCID xenograft mice were treated after large plasmacytomas had been established. EDO-S101 prolonged OS when compared with placebo and reduced further tumor growth. Immunohistochemical analysis of tumor tissue showed an increase in gamma-H2AX foci. Results were subsequently validated in the Vk*MYC MM mouse model, although only a low number of mice were used. Interestingly, a significant OS improvement was also seen in the very aggressive and multidrug-resistant Vk12653 model. These findings thus suggest that EDO-S101 could be a possible compound for treating patients with RRMM, and a phase I study is currently actively investigating this compound in this setting (NCT02576496).

Homologous recombination (HR) is an important DNA repair mechanism. Another set of key enzymes in the maintenance of the genome are PARP1 and PARP2. These proteins are required for repair of single-start breaks and inhibition of PARP1/2 causes a collapse of the DNA replication fork and formation of DSBs. As such, a combinational inhibitory strategy could induce so called synthetic lethality. Possible targets of HR include the cyclin-dependent kinases (CDK), which are upstream regulators of the HR pathway (Aylon et al., 2004). Alagpulinsa et al. (2016) adopted this approach and evaluated dinaciclib, a CDK inhibitor, in MM. They saw that exposure of different human MM cell lines abrogated HR repair and sensitized the cells to PARP inhibition using ABT-888, a PARP1/2 inhibitor, thereby inducing synthetic lethality. *In vivo* experiments once again underline the added benefit of murine mouse models to validate *in vitro* findings. CB-17/SCID mice were injected with RPMI8226 cells and tumor size was evaluated approximately 14 days post-inoculation. Mice were randomized into four groups to receive either placebo, dinaciclib, ABT-888, or combination therapy. The obtained *in vitro* results could be reproduced *in vivo* as mice treated with both compounds had less tumor growth and a longer survival when compared with monotherapy with either compounds. Lastly, transcriptional analysis of excised tumor tissue was also able to show significant downregulation of Rad51 and Brca1 mRNA levels.

### Study of Clonal Selection Using Murine MM Models

Over the last years, many novel insights concerning the presence and the importance of intratumor heterogeneity in cancer have become apparent. This biological concept is especially important when looking at MM. Although the initiating event in MM are clonal in nature, additional driver lesions can occur in specific subpopulations, or subclones, of cells. This biological concept can thus facilitate subclonal selection during treatment, relapse, and eventually the development of RRMM. (Lohr et al., 2014; Rasche et al., 2017; Corre et al., 2018). Generally, three subtypes of clonal selection have been identified in MM, being i) the presence of a stable, unchanged clonal architecture, ii) linear clonal evolution, and iii) a branching evolution of the clonal architecture, which is most often seen at relapse and displays a higher degree of genomic instability (Bolli et al., 2014; Weinhold et al., 2016). Another layer of complexity is added due to an increasing body of data suggesting the presence of important fluctuations in clonal architecture during treatment and relapse (Keats et al., 2012).

Melchor et al. (2014) used a NOD/SCID xenograft model, using CD138+ cells from a patient with plasma cell leukemia harboring four distinct tumor clones at time of diagnosis, to evaluate the presence of these clonal fluctuations and to study clonal phyologeny in greater detail. Xenograft tumor DNA was analyzed using whole-exome sequencing and was compared to tumor exomes from the patient samples at time of diagnosis and first relapse. Using this strategy, Melchor et al. (2014) wanted to analyze whether the different clonal types would compete with each other and would respond differently to the given treatment. Interestingly, they were able to demonstrate that different clones exhibit distinct survival and proliferating strategies following patient treatment or xenotransplantation and that early clones that were present at nearly undetectable levels at diagnosis were able to survive treatment and contribute to patient relapse. These important observations could also be partly confirmed in the xenograft model, thus solidifying the observed clonal fluctuations and the interclonal differences in withstanding selective pressure under the form of treatment or xenotransplantation.

Another prime example of using murine models to study clonal architecture in MM was provided by Hewett et al. (2017). They used the 5TMM model to study the efficiency of plasma cell migration toward, and the capability to grow, within the bone marrow niche. They tagged 5TGM1 cells *via* transfection of degenerate DNA barcodes after which the cells were injected into C57BL/KaLwRij mice. They were able to show that MM cells displayed important differences in growth capabilities and that clones that are few in numbers can eventually give rise to the bulk of the disease burden. This observation suggests that many MM cells remain dormant and that those that migrate to the bone marrow are subjected to an important selection pressure when they interact with the bone marrow niche, as this observation was not seen *in vitro*. This concept of *in vivo* clonal competition could also be seen in the Vk*Myc mouse model (Keats et al., 2012). Aged Vk*MYC mice were identified that developed bi- or triclonal MM disease, which showed dynamic changes in clonal distribution without specific therapeutic intervention. Subsequent transplantation of bone marrow cells from Vk*MYC mice with biclonal M-spikes into congenic C57BL/6 wild-type mice showed that only one of both clones was able to engraft. Secondly, bone marrow cells from Vk*MYC mice with either an aggressive or indolent MM were transplanted in parallel into wild type and Vk*MYC mice with pre-existing MM disease, which was distinct from the graft. Using this approach, Keats et al. were able to show that the most aggressive Vk*MYC clones engrafted more rapidly in congenic wild type mice, were also able to engraft in Vk*MYC tumor bearing mice, and were able to completely replace the pre-existing MM clone. As indolent MM cells were not able to engraft in mice with pre-existing MM and took longer to engraft in wild-type mice, these experiments validated the concept of clonal variation and possible dominance. Selective pressure on subclones through therapeutic intervention could also be seen in this murine model as treatment with novel agents leads to the selection and progression of refractory clones.

## Conclusion

Even in the era of novel drugs, certain MM subclones remain resistant to therapeutic interventions and will lead to relapse. Our current understanding of MM has evolved enormously and novel concepts, such as the involvement of genomic instability and its influence on drug resistance and clonal selection, leading to dismal patient outcomes have become very important in the field. As such, translational research focusing on the further elucidation of these aspects of MM is of the utmost importance, as novel insights could lead to novel treatment modalities and thus improve both the quantity and quality of the lives of MM patients. As summarized in this review, murine models are one of the most valuable tools to validate experimental findings *in vivo*. Luckily, many different models are present that mimic human disease reliably. There is, however, an important remark that has to be kept in mind when using murine models in MM research, being the fact that the different models have a different mode of construction, as has been described in the first part of this review. As such, one has to select the type of murine model that is best suited for the specific aspect of MM pathophysiology that is being studied. Also, detailed genetic characterization of the MM component is not present for all models and needs to be taken into account in view of the research question at hand. As such, thorough genetic analysis of the transgenic MM models would generate valuable additional insights into the pathophysiology of these widely used models, broadening our view on the mechanisms by which the murine MM models mimic human disease. Moreover, generation of additional transgenic MM murine models containing mutations of other specific MM subtypes, such as t(4;14)/MMSET, del(17p)/p53, or t(11;14)/CCND1, would be of great interest. Concerning the xenograft models, recent data covering the genetic characterization of human MM cell lines have been published, showing that specific MM cell lines do indeed exhibit deregulation of key pathways including DNA damage response and repair pathways, making them interesting tools to study DNA repair or genomic instability and as such validate potential therapeutic agents (Tessoulin et al., 2018; Vikova et al., 2019). Despite these observations, the use of murine models remains of the utmost importance if we are willing to advance the field as they generate novel mechanistic insights into genomic instability. Using murine models also facilitates the identification of potential therapeutic targets, such as those involved in drug resistance or high-risk disease. Many of these targets, exemplified in this review by mIR-137 and TRIP13, are, however, not fully studied in primary human MM samples. Additional research on the correlation between murine and human data, pertaining to disease risk characteristics or potential therapeutic responses, is therefore needed to be able to successfully translate murine *in vivo* findings into clinical practice. As also shown in the second part of this review, pre-clinical evaluation of experimental treatment strategies or toxicity and efficacy of novel compounds can only be performed *in vivo* and is needed to decide whether a promising compound can be withheld for evaluation in a first-in-human clinical trial. Moreover, aspects such as clonal evolution can only be understood to the fullest when using murine models.

## Author Contributions

PV, KV, and KM designed the outline of the manuscript, performed literature research, and wrote the manuscript. KDV, EM, EDB, FO, and KV critically revised the manuscript for intellectual content.

## Funding

This work was funded by FWO-Vlaanderen, International Myeloma Foundation and Vrije Universiteit Brussel under the strategic research program scheme “SRP48”. KM and KDV are postdoctoral fellows FWO-Vlaanderen under grants “12E3819N” and “12I0917N”.

## Conflict of Interest Statement

The authors declare that the research was conducted in the absence of any commercial or financial relationships that could be construed as a potential conflict of interest.
